# Machine Learning Predictive Models for Survival in Patients with Brain Stroke

**DOI:** 10.34172/hpp.025.43635

**Published:** 2025-05-06

**Authors:** Solmaz Norouzi, Samira Ahmadi, Shayeste Alinia, Farshid Farzipoor, Azadeh Shahsavari, Ebrahim Hajizadeh, Mohammad Asghari Jafarabadi

**Affiliations:** ^1^Student Research Committee, Faculty of Medical Sciences, Tarbiat Modares University, Tehran, Iran; ^2^Social Determinants of Health Research Center, Health and Metabolic Diseases Research Institute, Zanjan University of Medical Sciences, Zanjan, Iran; ^3^Department of Statistics and Epidemiology, Faculty of Medicine, Zanjan University of Medical Sciences, Zanjan, Iran; ^4^Department of Health Education and Promotion, Faculty of Health, Tabriz University of Medical Sciences, Tabriz, Iran; ^5^Department of Computer Engineering, Faculty of Engineering, Shabestar Branch, Islamic Azad University, Shabestar, Iran; ^6^Department of Biostatistics, Faculty of Medical Sciences, Tarbiat Modares University, Tehran, Iran; ^7^Cabrini research, Cabrini health, Melbourne, VIC, 3144, Australia; ^8^School of Public Health and Preventive Medicine, Faculty of Medicine, Nursing and Health Sciences, Monash University, Melbourne, VIC, 3004, Australia; ^9^Department of Psychiatry, School of Clinical Sciences, Faculty of Medicine, Nursing and Health Sciences, Monash University, Melbourne, VIC, 3168, Australia

**Keywords:** Brain stroke, Cox model, Machine learning algorithms, Prediction, Survival

## Abstract

**Background:**

This study aims to harness the predictive power of machine learning (ML) algorithms for accurately predicting mortality and survival outcomes in brain stroke (BS) patients.

**Methods:**

A total of 332 patients diagnosed with BS were enrolled in the study between April 21, 2006, and December 22, 2007, and then followed for 15 years (until 2023). Mortality outcomes were modeled using various statistical techniques, including the Cox model, decision trees, random survival forests (RSF), support vector machines (SVM), gradient boosting, and mboost. The best-performing model was selected based on diagnostic performance metrics: specificity, sensitivity, precision, accuracy, area under the receiver operating characteristic curve (AUC), positive likelihood ratio, negative likelihood ratio, and negative predictive value.

**Results:**

The results indicate that ML models in small sample sizes, particularly the SVM, outperformed the Cox model in predicting mortality and survival over 15 years, achieving an accuracy of 85% and an AUC of 0.765 (95% CI 0.637-0.83). Furthermore, the study identified important variables, including blood pressure history, waterpipe smoking, lack of physical activity, type of cerebrovascular accident, current smoking status, sex, and age, which provide valuable insights for clinicians in risk assessment.

**Conclusion:**

Our study showed that the SVM model outperforms the Cox model in predicting 15-year mortality and survival, particularly in small sample sizes. Moreover, the identification of key risk factors such as blood pressure history, waterpipe smoking, lack of physical activity, type of cerebrovascular accident, current smoking status, sex, and age highlights the need for their consideration in clinical assessments to enhance patient care.

## Introduction

 Brain stroke (BS) is a significant health problem.^1^ It is a neurological condition caused by either ischemic or hemorrhagic brain arteries, often resulting in motor and cognitive impairments that impact functionality.^2^ Approximately 16 million individuals worldwide experience BS yearly, leading to substantial societal costs. The high mortality rate associated with BS further emphasizes its severity as a health issue, as recognized by the American Heart Association.^3^ Additionally, the cost of hospitalization for BS is rising.^4^ Consequently, there is a growing need for advanced technologies to aid in clinical diagnosis, treatment, and event prediction.^5^ Several risk factors associated with BS have been identified in affected individuals. Comprehending risk determinants is essential for formulating plans for evidence-backed BS care, and judicious allocation of resources while confronting these risks with dedicated interventions and screenings is imperative for preventive measures.^6^

 Traditional methods for predicting the survival of patients are based on existing clinical predictors, using Cox regression analysis.^7^ It is extensively used in clinical research due to its wide applicability and ability to handle various survival time distributions. However, the Cox regression assumes that the mortality risk for different individuals remains constant over time, a condition that is often not met in real-world situations. Consequently, the Cox regression may not provide the best fit for each dataset. Furthermore, while the Cox regression has advantages as a linear model, it fails to express the complex nonlinear relationship between the logarithmic risk ratio and covariates.^8^

 Recent research has increasingly utilized machine learning (ML) methods to predict stroke outcomes and identify patients who could benefit from specific rehabilitation therapies.^9^ Skilled at handling multiple variables, these methods are particularly suited for complex conditions like stroke, eliminating the need for preprogrammed rules.^10-12^ ML methods, adept at analyzing vast datasets and complex patterns, have proven to be as or more effective than traditional models, such as the Cox regression in forecasting stroke outcomes and patient survival. Bandi et al^13^ utilized ML for the prediction of BS severity in their 2020 article. Furthermore, Rahman et al^9^ provided a study on predicting BS using ML algorithms and deep neural network techniques. Tazin et al^14^ and Krishna et al^15^ also applied an ML algorithm for BS. By improving precision and efficiency in survival analysis, ML holds significant potential for early stroke detection, a crucial step for effective treatment, making it one of the most effective technologies for health professionals in making clinical decisions and predictions.^16-20^

**Figure 1:**
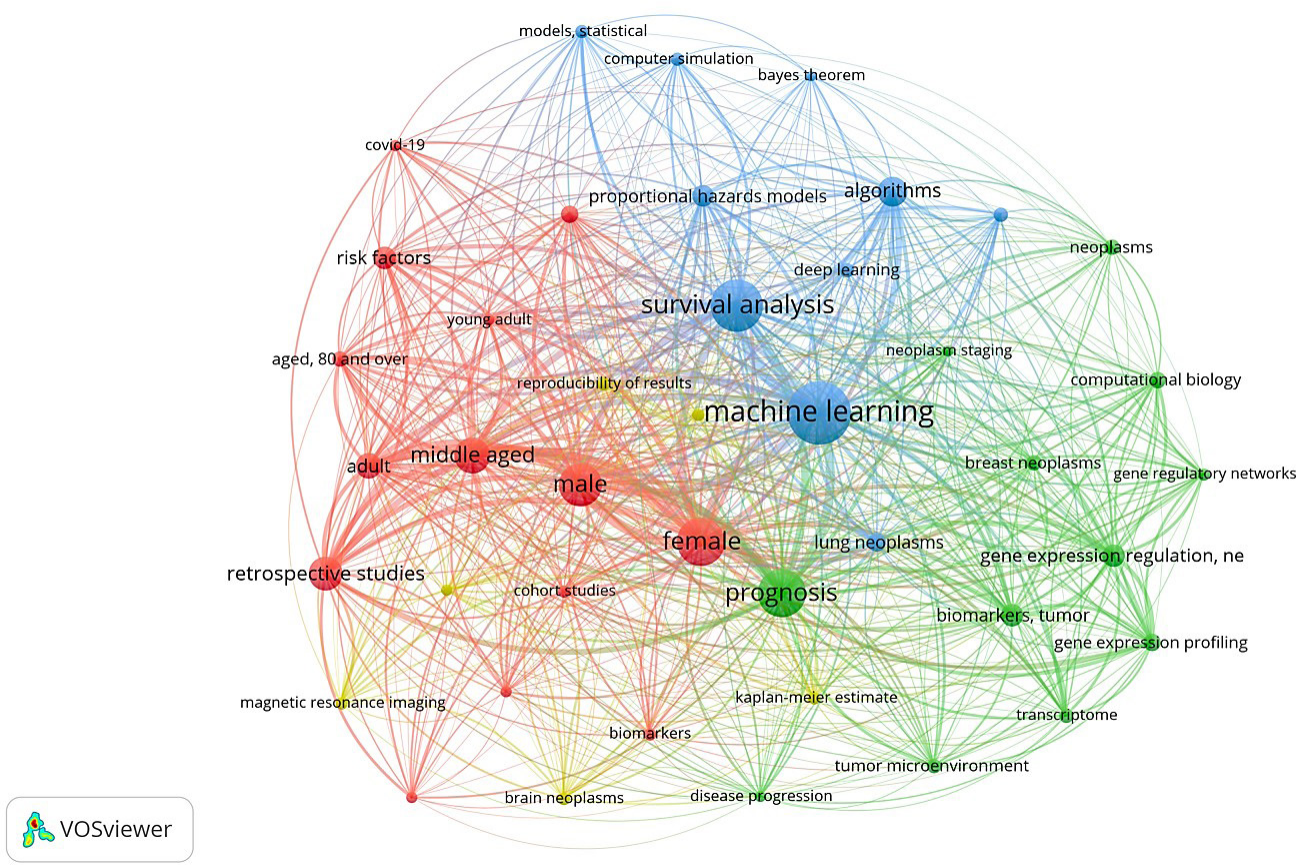
Co-occurrences of keywords of brain stroke mortality modeling. Red represents Cluster 1, green represents Cluster 2, blue represents Cluster 3, and yellow represents Cluster 4

 We chose VOSviewer for its ability to visualize scientific networks and identify clusters. Using this software, we explored the relationship between ML and survival analysis. By visualizing and analyzing various research themes, our goal was to enhance our understanding of how ML techniques affect survival outcomes in medical contexts. Four main clusters were identified: risk factors and treatment outcomes in COVID-19, gene expression profiling and prognosis in cancer, ML applications in lung cancer modeling, and imaging techniques for prognostic prediction in brain cancer. However, there have been very few studies focusing on the survival of stroke patients (Figure 1).

 In this study, we employed multiple ML algorithms alongside the Cox model to enhance the prediction of survival rates in patients with BS, particularly in a setting with a limited sample size. By evaluating performance metrics such as sensitivity, specificity, and area under the receiver operating characteristic curve (AUC), our goal was to identify the most accurate predictor for BS patient survival. We compared the established Cox regression model with various ML techniques, including decision trees (DT), random survival forests (RSF), support vector machines (SVM), gradient boosting (GB), and Model-Based Boosting (mboost). This approach has the potential to significantly improve clinical practice by providing more precise prognostic insights for BS patients.

## Materials and Methods

###  Study population

 The study population consisted of 332 patients with BS in Ardabil Province, Iran, within the period from April 21, 2006, to December 22, 2007. These patients were followed up from the beginning of 2008 to 2023. These patients were enrolled from Imam Khomeini Hospital, Ardabil, and were followed up for 15 years from the time of diagnosis. During this period, patients either died due to BS or other causes or survived. Data were extracted from the patient’s medical records.

 Eligibility for the study was limited to patients who were experiencing their first BS and had voluntarily agreed to participate after being informed about the study. The researchers used the ICD-10 diagnostic codes to classify all participants’ stroke diagnoses, based on the results of their CT and MRI scans. Demographic data and information on major clinical risk factors were extracted from the patient’s hospital records and incorporated into the analysis.

 The researchers determined the patients’ outcomes by contacting their family members. For those participants who passed away during the study period, the exact date and cause of death were documented and examined as part of the analysis. Patients with a previous history of BS or transient ischemic attack, as well as those with incomplete information in their medical records or who did not receive any treatment, were excluded.

###  Feature extraction 

**Figure 2:**
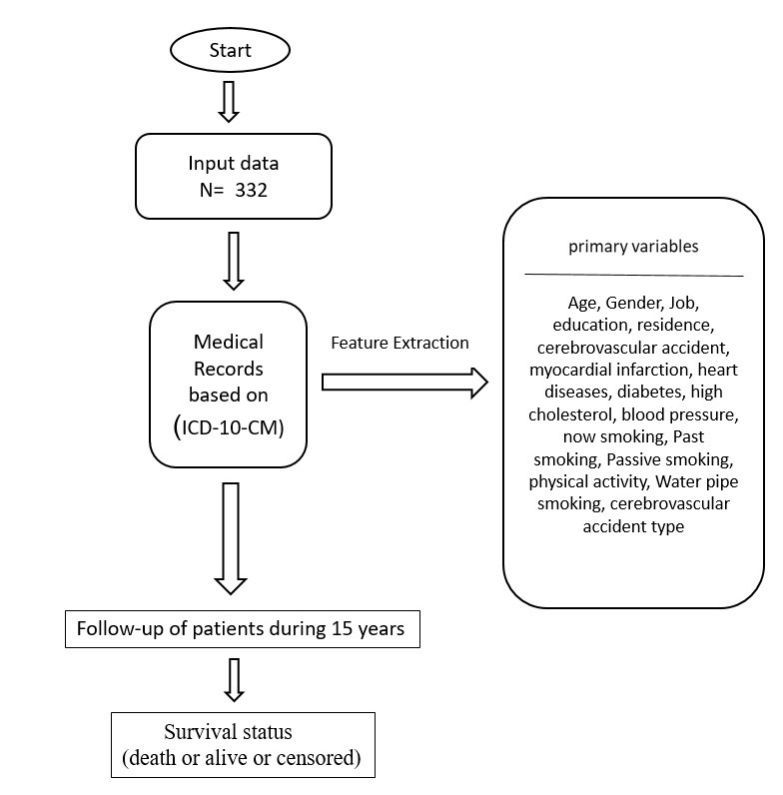
Feature extraction based on the brain stroke scale. Age of the patient at the time of diagnosis (years) ( ≤ 58, 59-68, 69-75, ≥ 76), gender of the patient (Male, female), Category of patient’s job status (employed, unemployed), category of patient’s education level (educated; uneducated), the patient's residence (urban area or rural), a history of cerebrovascular accident (yes, no), history of myocardial infarction (yes, no), heart disease (yes, no), diabetes disease (yes, no), history of high cholesterol (yes, no), blood pressure history (yes, no), now smoking (yes, no), past smoking (yes, no), Passive smoking (yes, no), physical activity (yes, no), waterpipe smoking (yes, no), cerebrovascular accident type (ischemic, hemorrhagic), time duration of follow-up in years (15 years), survival status (dead or alive or censored)

 Variable selection is crucial for robust and clinically meaningful analyses. So, we have selected a comprehensive set of demographic, clinical, and lifestyle factors associated with BS risk and prognosis. Clinical and demographic variables for all patients were analyzed using hospital records. Figure 2 illustrates the process of feature extraction based on the BS Scale.

###  Statistical analysis

 Statistical analysis was performed with R software [ver.4.3.2] (http://www.r-project.org/). In this research, we utilized R software packages like e1071, pROC, caret, rpart, party, ranger, survival, gbm, xgboost, survminer, and survivalsvm. Survival time was calculated in months, and the mean survival time (with its 95% confidence interval [CI]) is reported. The log-rank test was used to compare survival probabilities between groups.

 We used several ML algorithms for were assessed to predict the survival of patients with BS. DT, hierarchical models based on decision rules, are suitable for smaller-scale problems due to their interpretability.^20^ RSF, a variant of Random Forests, excel in complex survival analysis tasks and improve the handling of censored data. GB, combines weak models to form a stronger one and iteratively optimizes the objective function, typically using simple base functions like decision trees.^20,21^ SVM are used for classification and regression tasks, and in survival analysis, they are adapted to handle survival data, accounting for censored observations.^22^

 The principle of ML involves using data to predict an output based on a set of features or variables. In this study, supervised learning was employed due to the nature of the data, which involved predicting mortality due to BS. This type of learning involves computer learning from a dataset with labeled outcomes, such as whether the patient died or not.

 The primary outcome of our study is mortality due to BS. To enhance the precision of our results, we employed two dependent variables, time and event, concurrently for data analysis.

- Event: This is a binary variable indicating whether the event of interest (death due to stroke in our case) occurred during the study period. It is coded as 1 if the event occurred and 0 if it did not (i.e., the patient was censored).
- Time: This variable represents the time elapsed from the start of follow-up (diagnosis) to either the occurrence of the event (death) or the end of the observation period (end of follow-up or censoring).

This approach allows us to discover more complex patterns and more accurately examine the relationship between death factors and survival time.^23^ In cases where patients died due to causes other than stroke, censoring was applied. This means that their survival time up to the time of death was considered as censored data (competing risk censoring).

 Before building a predictive model using ML, it is crucial to clean the data. Therefore, we performed data cleaning, which included removing duplicates, correcting errors, filtering outliers, handling missing data, and dealing with censored observations. This procedure guarantees the accuracy and appropriateness of the data for creating successful predictive models. The next step is to train the model by dividing the dataset into two parts. The majority portion, typically approximately 80%, is used to train the model. During this process, the chosen algorithm analyzes the patterns present in the data and learns from them. The remaining portion of the dataset is then used to test and validate the model.

###  Model performance in models predicting a binary outcome

 Classification models are commonly assessed using the AUC parameter, which measures the discriminatory ability of a model. A higher AUC, usually above 0.70, indicates good discriminatory ability, while values below 0.50 suggest a lack of discriminatory ability.^24^ Apart from the AUC, model performance can be evaluated using a confusion matrix. This matrix allows for the calculation of various metrics, including accuracy, precision, sensitivity, and specificity.^25^ These metrics provide a comprehensive assessment of the model’s performance. Sensitivity measures the number of correctly identified positive cases as a proportion of the total positive cases. Specificity is calculated as the total number of accurate negative identifications divided by the total number of actual negative instances. Precision yields several positive outcomes when distinguished by a variety of positive results identified by the classifiers.^25^ In this study, we employed a time-to-event-dependent analysis.

## Results

###  Characteristics of the population

Table 1 presents the demographic and clinical characteristics of the participants. Among the patients, 26.8% were aged 58 or younger ( ≤ 58) and 19% were 76 or older ( ≥ 76). Additionally, 50.6% were male, 88% were employed, 74.7% had no academic education (below a diploma), and 60.5% resided in urban areas.

**Table 1:** Participants’ demographic and clinical characteristics

**Feature**		**N (%)**	**mortality rate (per 1000) (95%CI)**	***P *** **value**
Age (y)	≤ 58	89 (26.8)	10.93 (8.02-14.90)	< 0.005
	59-68	83 (25)	17.08 (13.08-22.31)	
	69-75	97 (29.2)	20.62 (16.58 -25.64)	
	≥ 76	63 (19)	27.96 (21.31-36.69)	
Gender	Female	164 (49.4)	16.74 (13.81-20.29)	0.010
	Male	168 (50.6)	19.24 (16.12-22.96)	
Job status	Employed	291 (87.7)	17.45 (15.12-20.13)	< 0.005
	Unemployed	41 (12.3)	21.29 (15.55-29.14)	
Education level	Educated	84 (25.3)	18.59 (14.17-24.40)	0.121
	Uneducated	248 (74.7)	17.84 (15.38-20.69)	
Residence	Urban	201 (60.5)	16.84 (14.25-19.91)	0.254
	Rural	131 (39.5)	20.12 (16.36-24.74)	
A history of cerebrovascular accident	Yes	80 (24.1)	19.02 (14.24-25.39)	0.031
	No	252 (75.9)	17.76 (15.36-20.55)	
History of myocardial infarction	Yes	24 (7.2)	14.86 (9.10-24.26)	0.444
	No	308 (92.8)	18.30 (15.99-20.94)	
Heart disease	Yes	86 (25.9)	21.52 (16.81-27.55)	0.109
	No	246 (74.1)	16.94 (14.54-19.74)	
Diabetes disease	Yes	60 (18.1)	25.76 (19.23-34.50)	0.050
	No	272 (81.9)	16.76 (14.49-19.38)	
History of high cholesterol	Yes	62 (18.7)	16.87 (12.23-23.29)	0.047
	No	270 (81.3)	18.24 (15.83-21.03)	
Blood pressure history	Yes	197 (59.3)	18.78 (15.96-22.06)	0.003
	No	135 (40.7)	16.72 (13.41-20.84)	
Now smoking	Yes	64 (19.3)	19.49 (14.40-26.37)	0.998
	No	268 (80.7)	17.70 (15.33-20.44)	
Past smoking	Yes	95 (28.6)	16.07 (12.69-20.34)	0.276
	No	237 (71.4)	19.01 (16.26-22.22)	
Passive smoking	Yes	59 (17.8)	18.01 (13.21-24.55)	0.692
	No	273 (82.2)	18.01 (15.60-20.78)	
Physical activity	Yes	46 (13.9)	16.77 (11.58-24.28)	0.198
	No	286 (86.1)	18.19 (15.83-20.91)	
Water pipe smoking	Yes	11 (3.3)	16.80 (9.04-31.24)	0.140
	No	321 (96.7)	18.07 (15.81-20.64)	
Cerebrovascular accident type	Ischemic	266 (80.1)	16.48 (14.23-19.10)	0.026
	Hemorrhagic	66 (19.9)	26.75 (20.28-35.30)	

Mortality rate = failures/person-time (per 1000), and the results of tests comparing the rates. Bold p values indicate significant differences (*p* < 0.05).

**Figure 3:**
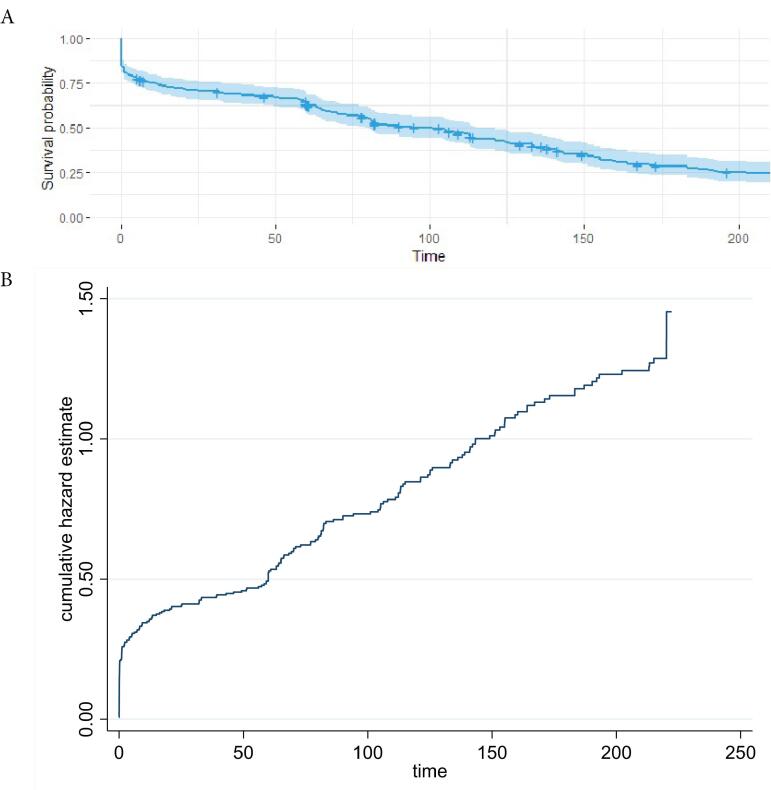
(A) The probability of survival of brain stroke patients over time, (B) Nelson Aalen cumulative hazard estimate

 A total of 332 patients with BS were followed up for 15 years, and the mortality rate due to BS was 68.4%. Patients who died due to BS had a mean survival time of 55.52 months ( ± 4.14) and the median survival time was 32.17 months. The 5-year, 10-year, and 15-year survival rates were 60.96% (95% CI: 55.47-65.99), 42.72% (95% CI: 37.20-48.12), and 27.44% (95% CI: 22.41-32.69), respectively (Figure 3). The line graph depicts the likelihood of survival over time, indicating a decline in the probability of survival for patients with BS.

###  Mortality rates

 The mortality rate for BS increased significantly with age, and males had higher rates than females. Unemployed individuals also experienced greater mortality compared to employed ones. BS-related deaths were more prevalent among patients with a history of cerebrovascular accidents, diabetes, high cholesterol, and hypertension. Other factors did not significantly affect BS mortality (*P* < 0.05) (see Table 1 for further details).

###  Performance evaluation of ML models and Cox model

Table 2 shows the sensitivity, specificity, accuracy, precision, and AUC ROC scores of the five classifiers on the 15-year test data. Accuracy measures the percentage of correctly classified instances, while AUC-ROC assesses the classifier’s ability to distinguish between dead and alive patients.

**Table 2:** Comparison of the Cox model and five machine learning algorithms for brain stroke mortality using test data

**Outcome**	**Model**	**Sensitivity** **(95% CI)**	**Specificity** **(95% CI)**	**Accuracy (95% CI)**	**Precision** **(95% CI)**	**AUC** ** (95% CI)**	**LR+** **(95% CI)**	**LR-** **(95% CI)**	**NPV** **(95% CI)**
Brain stroke mortality (15 years)	SVM	0.68 (0.58, 0.76)	0.94 (0.90, 0.97)	0.86 (0.82, 0.89)	0.85 (0.75, 0.91)	0.765 (0.637-0.83)	11.81 (6.85, 20.35)	0.34 (0.26, 0.45)	0.86 (0.81, 0.90)
	Cox model	0.61 (0.48, 0.73)	0.75 (0.70, 0.80)	0.73 (0.67, 0.77)	0.36 (0.27, 0.46)	0.758 (0.70, 0.81)	2.47 (1.85, 3.29)	0.51 (0.37, 0.71)	0.89 (0.85, 0.93)
	mboost	0.47 (0.37, 0.57)	0.82 (0.77, 0.87)	0.71 (0.66, 0.76)	0.55 (0.44, 0.66)	0.694 (0.63 – 0.75)	2.65 (1.87, 3.75)	0.65 (0.54, 0.78)	0.77 (0.71, 0.82)
	DT	0.83 (0.36, 1.00)	0.66 (0.52, 0.77)	0.67 (0.55, 0.78)	0.19 (0.07,0.39	0.763 (0.63-0.88)	2.42 (1.47, 3.98)	0.25 (0.04, 1.54)	0.98 (0.87, 1.00)
	GB	0.69 (0. 48, 0.86)	0.62 (0.46, 0.77)	0.65 (0.52, 0.76)	0.55 (0.36, 0.72)	0.733 7(0.55, 0.78)	1.85 (1.15, 2.97)	0.49 (0.26, 0.92)	0.76 (0.58, 0.89)
	RSF	0.21 (0.09, 0.39)	0.42 (0.25, 0.61)	0.32 (0.21, 0.44)	0.27 (0.12, 0.48)	0.750 (0.57-0.80)	0.37 (0.18, 0.76)	1.86 (1.20, 2.87)	0.35 (0.21, 0.52)

The optimal model is shown in boldface font. The measures are estimated in the test dataset. SVM, Support vector machine; GB, gradient boosting; DT, Decision tree; RSF, random survival forest; AUC, area under the curve; LR + , positive likelihood ratio; LR-, negative likelihood ratio; NPV, negative predictive value; CI, confidence interval.

**Figure 4:**
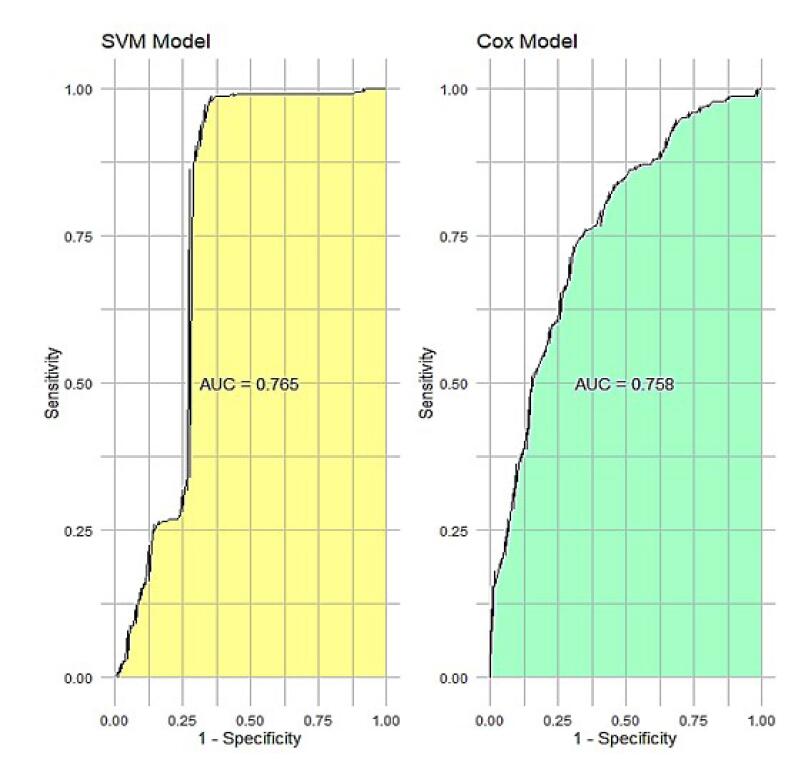
Two ROC plots side by side for the two best models on test data

- Accuracy: SVM achieves the highest average accuracy score of 0.86. This indicates that, on average, SVM correctly classifies approximately 86% of the instances in the dataset. This was followed by a Cox model of 0.73 and an m boost of 0.71. DT, GB, and RSF demonstrated lower average accuracy scores of 0.67, 0.65, and 0.32 respectively (Table 2).
- ROC AUC: SVM had the highest average ROC AUC of 0.765. A ROC AUC of 1.0 represents a perfect classifier, so the SVM score indicates a strong ability to distinguish between patients who died and those who survived. DT, Cox model, RSF, GB, and m boost exhibited lower average ROC AUC values of 0.763, 0.758, 0.750, 0.733, and 0.694, respectively (Table 2 and Figure 4).

###  SVM feature selection and Cox regression analysis

**Figure 5:**
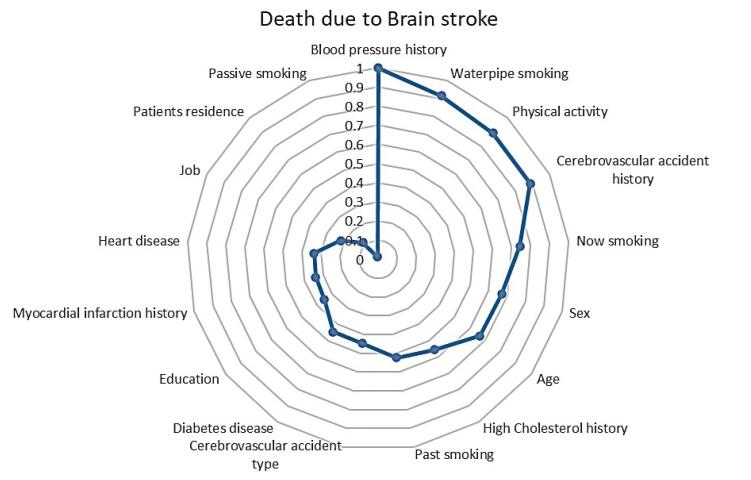
Radar plot for the best model selection based on the significant features for predicting brain stroke (BS) mortality using support vector machines (SVM)

Figure 5 presents a radar plot summarizing the predictive significance of various features for BS mortality using the SVM model. Based on the results, it seemed that blood pressure history was the most importance predictor, followed by waterpipe smoking, physical activity, and cerebrovascular accident history, which were the most influential predictors as indicated by their proximity to the outermost circle.

 In this section, after selecting the most important variables using the best model (SVM), the hazard ratio (HR) of each variable was calculated by applying the Cox model.

 Based on the optimal model SVM, the significant features for predicting BS mortality are blood pressure history (HR = 1.51, 95% CI = 1.14-1.98), waterpipe smoking (HR = 1.60, 95% CI = 0.85-3.02), not physical activity (HR = 1.29, 95% CI = 0.87-1.92), cerebrovascular accident type (HR = 1.42, 95% CI = 1.01-2.02), now smoking (HR = 1.01, 95% CI = 0.71-1.41), Sex (HR = 1.40, 95% CI = 1.08-1.83), and age (59-68 year (HR = 2.27, 95% CI = 1.50-3.42), 69-75 year (HR = 3.92, 95% CI = 2.67-5.78), ≥ 76 year (HR = 4.62, 95% CI = 3.03-7.05)). So patients over 70 years are at a higher risk of BS mortality than those under 58 years.

## Discussion

 In this study, we conducted a 15-year follow-up of 332 patients diagnosed with BS, revealing a mortality rate of 68.4%. The mean survival duration for those who succumbed to BS was approximately 55.52 months, with a 15-year survival rate of 27.44% (95% CI: 22.41% - 32.69%). Our findings demonstrate a progressive decline in survival over time.

 Log-rank test findings highlight key factors associated with increased mortality in BS patients. The rise in mortality with age aligns with age being a major risk factor for cardiovascular and cerebrovascular diseases, which can worsen BS complications. Higher mortality in males compared to females reflects increased vulnerability in men, influenced by both biological and lifestyle factors. The elevated mortality in unemployed individuals emphasizes the impact of socioeconomic factors, such as limited healthcare access and unhealthy behaviors, on health outcomes.^26,27^ A strong association between BS-related deaths and comorbidities like cerebrovascular accidents, diabetes, high cholesterol, and hypertension.^28-30^ underscores their compounded effect on mortality risk. While other factors did not show significant associations, further research is needed to explore these relationships in greater detail.

 When comparing predictive models, the SVM outperformed the Cox model in forecasting 15-year survival, achieving an accuracy of 86% and an AUC of 0.765 (0.637-0.83). After choosing SVM as the best model, important variables were extracted from the BS data. This step not only helps us identify key variables but also reduces the complexity of the model and increases the accuracy of predictions. Next, the HR for each variable was calculated using the Cox model. The Cox model, known as a regression model for survival analysis, enables us to examine the effect of each variable on the survival time or the occurrence of a specific event. By calculating the HR, we can better understand the relationship between variables and the desired outcomes.

 This study underscores the effectiveness of ML models in accurately predicting long-term outcomes for BS patients, particularly in smaller datasets,^30,31^ which aligns with previous research demonstrating the superiority of ML algorithms like XGBoost over traditional regression methods such as Cox regression.^28,29^ Also, studies have shown that SVM is effective in classifying patients at high risk of stroke, often achieving competitive accuracy compared to DT and ANN.^31^ Moreover, integrating SVM with advanced algorithms like XGBoost has significantly enhanced predictive capabilities, with some models achieving impressive accuracies of up to 99%. This highlights the potential of combining SVM with innovative techniques to develop robust and reliable predictive models for assessing stroke risk in patients.^32,33^ Furthermore, investigations into other medical conditions, including osteosarcoma, oral cancers, and renal cell carcinoma, further validate the advantages of ML models. These studies highlight the potential of ML approaches as robust alternatives in survival analysis, showcasing their ability to enhance predictive accuracy and improve clinical decision-making.^34,35^

 In a 2023 research study conducted by Rahman et al., the prediction of early-stage stroke occurrence was investigated using deep learning and ML methodologies. Interestingly, the RF classifier demonstrated a remarkable classification accuracy rate of 99%, surpassing that of the other ML classifiers. The empirical findings suggested that ML techniques outperformed deep neural networks in the specific context of stroke prediction.^9^

 The findings of this study identify several significant predictors that contribute to the risk of BS mortality, providing valuable insights for both clinical practice and future research.^36,37^ Key predictors include blood pressure history, waterpipe smoking, lack of physical activity, type of cerebrovascular accident, current smoking status, sex, and age.

 A history of high blood pressure is a risk factor for BS mortality, as elevated blood pressure can damage blood vessels and increase the likelihood of both ischemic and hemorrhagic strokes. This finding underscores the critical importance of regular monitoring and management of blood pressure, particularly in at-risk populations.^28-30^

 Additionally, the association of waterpipe smoking with BS mortality is noteworthy. While the detrimental health effects of traditional cigarette smoking are widely recognized, waterpipe smoking has often been underestimated. This study highlights the urgent need for increased awareness regarding the risks associated with waterpipe use, which may contribute to BS mortality.^38,39^

 Moreover, the lack of physical activity emerges as another critical predictor of stroke risk. Sedentary lifestyles are linked to various health issues, including obesity, hypertension, and diabetes, all of which can elevate BS mortality. Therefore, promoting physical activity should be a public health priority to significantly reduce stroke incidence.^26,27^

 Our findings underscore the consistent importance of age as a predictor of mortality in BS, aligning with previous research that has identified key predictors, including BMI and education level.^2,40-42^ These results are supported by systematic reviews (2022) and recent studies, which have highlighted the promise of ML techniques in various medical contexts, including cancer treatment and stroke prediction.^17,43-45^ Other studies have also indicated that advanced age,^46^ sex,^47,48^ BMI, and education level are important predictors of mortality in patients with BS.^37,49^ Targeting demographic factors in interventions can improve survival rates among patients with BS.

## Strengths and limitations

 The strengths of this study include its long follow-up period and the application of advanced predictive models, which deliver reliable and precise survival estimates across varying time intervals. However, the study has some limitations. The retrospective design may introduce inherent biases, and the results might not be generalizable to more diverse populations.

 Additionally, we have considered some confounders, but by incorporating stress levels and mental health, dietary habits, and type of treatment received variables into survival analyses, researchers can enhance predictive accuracy and ultimately improve patient care in clinical settings. Despite these limitations, the study significantly enhances our understanding of the predictors of mortality in BS patients and highlights the importance of personalized and timely interventions to improve patient outcomes.

## Conclusion

 This study utilized different ML methods to predict 15-year mortality due to BS. The SVM was identified as the optimal model for accurately predicting long-term survival in BS patients among the methods evaluated in this study. Our comprehensive analysis highlights the crucial significance of several factors in predicting long-term survival outcomes for patients with BS, including blood pressure history, waterpipe smoking, lack of physical activity, type of cerebrovascular accident, current smoking status, sex, and age.

 The superior performance of the SVM algorithm over traditional models like the Cox model, especially in handling small datasets, highlights the transformative potential of these advanced techniques in survival analysis. Moreover, the consistent identification of age and other key predictors across various studies reaffirms their importance in mortality prediction.

 These insights have significant implications for clinicians in terms of risk assessment and the development of targeted interventions to enhance patient care and improve outcomes. Predicting functional outcomes after a stroke is crucial for clinicians in setting reasonable goals with patients and relatives.

## Competing Interests

 The authors declare no competing interests.

## Ethical Approval

 This study was approved by the ethics committee of the School of Medical Sciences Tarbiat Modares University under the approval ID IR.MODARES.REC.1401.230. The participants’ privacy was preserved. All participants completed an informed consent form. All the processes were approved by international agreements (World Medical Association, Declaration of Helsinki, Ethical Principles for Medical Research Involving Human Subjects).
